# Improving Intensive Care Unit Nurses' Delirium Assessment Performance Through a Multimodal Educational Intervention

**DOI:** 10.1111/nicc.70168

**Published:** 2025-09-08

**Authors:** Rui‐Ling Chang, Shu‐Fen Siao, Shih‐Chi Ku, Yu‐Chang Yeh, Yu‐Chun Chang, Cheryl Chia‐Hui Chen

**Affiliations:** ^1^ Department of Nursing National Taiwan University Hospital and National Taiwan University College of Medicine Taipei Taiwan; ^2^ School of Nursing, National Taiwan University College of Medicine Taipei Taiwan; ^3^ Department of Internal Medicine National Taiwan University Hospital and National Taiwan University College of Medicine Taipei Taiwan; ^4^ Department of Anesthesiology National Taiwan University Hospital and National Taiwan University College of Medicine Taipei Taiwan; ^5^ Program in Occupational Therapy, Washington University School of Medicine in St. Louis St. Louis Missouri USA

**Keywords:** delirium, ICDSC, intensive care unit, nurse education

## Abstract

**Background:**

Delirium is a prevalent and serious ICU complication, particularly in elderly or ventilated patients. Accurate assessment is crucial but often inconsistent. Intensive care unit (ICU) nurses' use of the Intensive Care Delirium Screening Checklist (ICDSC) may be limited without structured training.

**Aim:**

To evaluate the delirium assessment performance of ICU nurses using ICDSC and assess the effectiveness of a multimodal educational intervention for performance enhancement.

**Study Design:**

This pre‐ and post‐intervention study was conducted in three medical ICUs in Northern Taiwan. The delirium assessment performance of ICU nurses using ICDSC was evaluated, followed by a three‐month multimodal educational intervention aimed at improving assessment performance. Each nurse's ICDSC assessment was paired with an independent assessment by a trained expert nurse. To ensure representation that reflects the true performance level, accounting for variations in nurses' working shifts, weekday and weekend staff ratios and sampling fairness across the three MICU units, the pairings were made using a three‐step randomization process, managed by an independent third party. A 3‐month multimodal educational intervention, including didactic lectures, difficult scenario reviews and one‐to‐one bedside mentoring, was implemented afterward. Inter‐rater agreement before and after the intervention was assessed using Cohen's kappa and Gwet's AC1 statistics.

**Results:**

The baseline agreement between ICU nurses and the expert nurse was suboptimal (kappa = 0.63, 95% CI: 0.57–0.70). The multimodal educational intervention was well‐received by the units, particularly among new nurses. Following the intervention, kappa significantly improved to 0.74 (95% CI: 0.69–0.80). Notable improvements were observed in key items of ICDSC, including the assessment of altered level of consciousness, inattention, disorientation, psychomotor agitation or retardation, and sleep–wake cycle disturbances. However, agreement remained poor for certain patient populations, especially those aged over 85 years and those subjected to physical restraint.

**Conclusions:**

A structured, multimodal educational intervention significantly improved the delirium assessment performance of ICU nurses using the ICDSC. One‐to‐one coaching and scenario‐based learning were particularly effective in enhancing clinical assessment skills. However, additional strategies may be required to address persistent challenges in assessing very elderly patients and those subjected to physical restraint.

**Relevance to Clinical Practice:**

A structured, multimodal educational intervention can substantially enhance the performance of ICU nurses in delirium screening using the ICDSC. Tailored training strategies may help bridge the knowledge–practice gap, leading to more reliable clinical assessments in critical care settings.

**Impact Statements**

- What is known about the topic
  ○ Delirium is a common and serious complication in ICU patients, especially older adults, medical ICU patients and those on mechanical ventilation. It leads to prolonged ventilation, extended ICU stays and long‐term cognitive issues, including a higher risk of dementia.
  ○ Timely delirium assessment is crucial for prevention and management. However, studies indicate that ICU nurses often perform suboptimally in delirium assessments before structured training, resulting in under‐recognition of the condition.
- What this paper adds
  ○ This study demonstrates that a structured, multimodal educational intervention can significantly improve ICU nurses' delirium assessment performance using the ICDSC.
  ○ The findings support the integration of tailored delirium education into routine ICU practice to promote more consistent and accurate bedside assessments.

## 1 Introduction

Delirium is a common and serious complication in patients in the intensive care unit (ICU), particularly among older adults [1], medical ICU patients [2, 3] and those receiving mechanical ventilation [3]. Its presence has been independently linked to prolonged mechanical ventilation and length of stay [4, 5], and long‐term cognitive impairment, including an elevated risk of dementia [6, 7]. Timely and accurate delirium assessment is critical for appropriate management and prevention [8]. However, studies have shown that ICU nurses' delirium assessment performance prior to formal training may be limited [9, 10, 11]. Although the Intensive Care Delirium Screening Checklist (ICDSC) is widely used for delirium assessment in ICU patients, its accuracy may vary depending on the nurse's experience and familiarity with the tool.

The ICDSC is a validated delirium assessment tool based on diagnostic criteria outlined in the Diagnostic and Statistical Manual of Mental Disorders (DSM). It evaluates delirium across eight dimensions: altered level of consciousness, inattention, disorientation, hallucinations or delusions, psychomotor agitation or retardation, inappropriate speech or emotional responses, disturbances in sleep–wake cycles and fluctuation in symptoms. Each dimension scores one point if abnormalities are observed, and a total score of ≥ 4 indicates delirium [12]. The ICDSC exhibits strong sensitivity and specificity (≥ 80%) with excellent inter‐rater reliability, effectively identifying delirium in critically ill patients [13].

## 2 Background

This study evaluated the performance of ICU nurses in assessing delirium using the ICDSC and examined the effectiveness of a targeted educational intervention. Each nurse's ICDSC assessment was compared with an independent evaluation by an expert nurse trained in ICDSC use. The expert nurse, experienced in all three ICUs, actively participated in patient care and handovers. We noted inconsistencies between assessment results and clinical presentations, prompting this quality improvement study. The lack of ICDSC assessment monitoring since the COVID‐19 pandemic further underscored the need for our initiative. On the basis of the baseline performance levels across three medical ICU units, we designed a multimodal educational intervention consisting of didactic lectures, case discussions of challenging scenarios and one‐on‐one bedside mentoring. This intervention was implemented at all three units. Its effectiveness was assessed by comparing pre‐ and post‐intervention agreement between nurses' ICDSC assessments (extracted from electronic medical records) and those of the expert nurse.

## 3 Research Question

What is the current delirium assessment performance of ICU nurses, as measured by agreement with an expert nurse using the ICDSC, and can a multimodal educational intervention enhance ICU nurses' assessment performance?

## 4 Design and Methods

This was a pre‐ and post‐intervention study conducted as part of the Delirium Management Initiative for medical ICU patients. The objective was to evaluate the effectiveness of a targeted educational intervention in improving ICU nurses' delirium assessment performance using the ICDSC. From December 2023 to February 2024, a multimodal, interactive educational intervention on delirium assessment was implemented across three ICUs by an independent expert nurse, who was a senior ICU clinician and had completed 4 weeks of specialized delirium training. The intervention included three components. First, didactic lectures equipped nurses with evidence‐based knowledge on delirium epidemiology, clinical manifestations, risk factors, prevention strategies and the standardized use of the ICDSC assessment tool. Second, case discussions addressed challenging scenarios identified through pre‐test data, which highlighted ICDSC items with higher error rates. In December 2023, the didactic lectures and case discussions were integrated into routine ward meetings, with two 30–60 min sections in each ICU, achieving participation rates of 80% or higher. In these interactive sessions, nurses collaboratively reviewed complex cases and engaged in focused discussions to refine their assessment techniques. Third, one‐on‐one bedside mentoring provided personalized, hands‐on instruction from an expert nurse. During these sessions, the expert nurse observed ICU nurses performing ICDSC assessments, offered immediate corrective feedback and reinforced proper techniques. During January and February 2024, each bedside nurse received two one‐on‐one mentoring sessions that included four instances of hands‐on practice with the ICDSC assessment. These sessions were scheduled according to individual shift rotations to ensure comprehensive coverage of all bedside nurses. This tailored educational approach addressed individual learning needs through direct observation and feedback, enhancing the assessment competence of each participating nurse.

### 4.1 Setting and Sample

The study was conducted at a 2600‐bed tertiary medical centre in Taiwan between September 2023 and May 2024. It involved three adult medical ICUs comprising a total of 41 beds and staffed by 104 nurses. Since 2019, these units have implemented standardized delirium assessment protocols using the ICDSC every 8‐h nursing shift as part of routine care.

### 4.2 Data Collection Tools and Methods

Each bedside nurse's ICDSC assessment was paired with an independent assessment conducted by an expert nurse trained in ICDSC use. Following a predefined randomization scheme, the expert nurse independently evaluated the assigned patient within 60 min of the bedside nurse's assessment. These evaluations occurred during designated shifts on both weekdays and weekends and included as many patients as feasible. Throughout the study, both the expert nurse and the bedside nurses remained blinded to each other's ICDSC results, thereby ensuring objectivity and preserving data integrity.

To ensure that the sample accurately reflected real‐world performance, a structured three‐step randomization process was implemented. First, to ensure all shifts were audited, each of the three daily shifts (day, evening and night) was randomly assigned to a different month. Second, within each month, the three MICU units were randomly ordered and each unit was allocated 1 week for auditing. Third, during each ICU's designated audit week, 4 days—3 weekdays and 1 weekend day—were randomly selected for data collection. All randomization procedures were managed by an independent third party to minimize bias and ensure sampling fairness across units, shifts and timeframes.

For pairing comparisons, each nurse's ICDSC assessment data were extracted from the electronic medical record by a third‐party research nurse who was blinded to the study protocol and had no direct involvement in the assessment process. This paired assessment procedure was conducted during both the baseline and post‐intervention phases, each lasting 3 months, forming a complete audit cycle. The post‐intervention assessments commenced within 2 weeks of intervention completion and were conducted from 1 March to 31 May 2024. Additionally, the ICDSC audit was delivered by an expert nurse who has extensive experience working in the same three ICU units and has received specific training for this role. The training included practical sessions with hands‐on ICDSC assessments, in‐depth case discussions and the resolution of any discrepancies in scoring. Usually, each assessment typically took 15–20 min and included a thorough review of the patient's medical records, noting recent changes in sedation regimens and relevant clinical notes from the current shift.

### 4.3 Data Analysis

Nurse performance in conducting ICDSC assessments was evaluated by comparing their ratings with those of a trained expert nurse using Cohen's kappa coefficient. This statistic measures inter‐rater agreement, with values interpreted according to Landis and Koch [14]: 0.21–0.40 as fair, 0.41–0.60 as moderate, 0.61–0.80 as substantial and > 0.80 as almost perfect agreement [14].

Kappa values were calculated for both the total ICDSC result (score ≥ 4/score < 4) and individual item‐level classifications, assessing whether bedside nurses correctly matched each patient's observed symptoms to the appropriate ICDSC category. The effects of the delirium educational intervention were assessed by comparing nurse performance before and after the intervention, as reflected by changes in kappa coefficients. Z‐tests were used to evaluate differences in pre‐ and post‐intervention kappa values, with statistical significance defined as *p* < 0.05.

In cases where the marginal distributions were symmetrical but unbalanced—meaning both raters disproportionately assigned responses to the same category—Cohen's kappa may underestimate agreement because of the kappa paradox [15]. Thus, Gwet's AC1, a statistic less affected by marginal imbalances [16], was also calculated as a supplementary index of agreement. For both kappa and AC1, 95% confidence intervals (CIs) were reported.

To explore patient‐related factors associated with lower nurse performance in ICDSC assessments (i.e., disagreements with expert ratings), multivariable logistic regression analyses were conducted separately for the pre‐ and post‐intervention phases. Independent variables included patient age categories, changes in RASS scores, mechanical ventilation status and use of physical restraints. Results were reported as odds ratios (ORs) with corresponding 95% CIs and *p*‐values, with *p* < 0.05 considered statistically significant.

Of note, the item ‘symptom fluctuation’ was excluded from item‐level analysis, as it was auto‐generated by the hospital system on the basis of prior shift records and not manually rated. However, it remained part of the total ICDSC score, and the primary outcome—overall delirium agreement (ICDSC score ≥ 4 vs. < 4)—included this item. In this study, overall agreement refers to the percentage of paired assessments where both the bedside nurse and expert nurse agreed on the presence or absence of delirium. It indicates the proportion of assessments where both scored either delirium (≥ 4) or no delirium (< 4). We report both overall agreement and the Kappa statistic: Kappa adjusts for chance agreement, although overall agreement provides a clearer measure of clinical concordance.

### 4.4 Ethical and Institutional Approvals

The study was approved by the Institutional Hospital Research Ethics Committee (IRB No. 202301153RINB) on 25 August 2023. A waiver of informed consent was granted for participating nurses, as the ICDSC is part of standard care and individual nurses were not treated as study participants; instead, the three medical ICU units were considered the units of analysis. The study was registered at the Clinical Trials Registry (Trial No.: NCT06054828).

## 5 Results

### 5.1 Overall and Subitem‐Level Accuracy Before Intervention

A total of 847 paired ICDSC assessments were analysed—420 before and 427 after the multimodal educational intervention. As presented in Table 1, the ICU nurse performance of delirium assessment before the intervention showed only moderate consistency, with a Cohen's kappa value of 0.63 (95% CI: 0.57–0.70), and overall agreement of 76%.

**TABLE 1:** Pre‐ and post‐intervention performance of ICDSC assessments.

	Bedside nurse					
	Pre‐intervention			Post‐intervention		
	No delirium	Delirium	Unable to assess^a^	No delirium	Delirium	Unable to assess^a^
Expert nurses						
No delirium	107	23	1	155	34	1
Delirium	35	129	13	16	138	5
Unable to assess^a^	3	26	8	6	15	57
	Agreement, % (*n*/*N*) = 76.0% (319/420) Cohen's kappa, (95% CI) = 0.63 (0.57–0.70)			Agreement, % (*n*/*N*) = 82.0% (350/427) Cohen's kappa, (95% CI) = 0.71 (0.66–0.77)		

Abbreviation: CI, confidence interval.

^a^ RASS = −4 to −5.

Among the eight subitems of the ICDSC (Table 2), prior to the intervention, levels of agreement—on the basis of the classification proposed by Landis and Koch [14]—varied across items. Psychomotor agitation or retardation (κ = 0.24) and sleep–wake cycle disturbance (κ = 0.29) exhibited only fair agreement. Altered level of consciousness (κ = 0.52) and inattention (κ = 0.48) achieved moderate agreement, whereas disorientation (κ = 0.68) demonstrated substantial agreement. In contrast, the items hallucinations and inappropriate speech or mood yielded low kappa values, which can be attributed to the kappa paradox, wherein skewed marginal distributions distort the kappa estimates despite high observed agreement [16]. Indeed, the raw agreement for these items was high—99.0% and 95.9%, respectively—indicating strong rater concordance. To address this discrepancy, Gwet's AC1 was further calculated and confirmed excellent pre‐intervention agreement (AC1 = 0.99 and 0.95). Thus, the low kappa values for hallucinations and inappropriate speech or mood were statistical artefacts rather than indicators of true disagreement.

**TABLE 2:** Summary of pre‐ and post‐intervention Cohen's kappa values.

Variables	Pre‐intervention	Post‐intervention	*p*
	Cohen's kappa (95% CI)	Cohen's kappa (95% CI)	
ICDSC results	0.63 (0.57–0.70)	0.71 (0.66–0.77)	**0.04**
Altered level of consciousness	0.52 (0.45–0.59)	0.80 (0.76–0.84)	**< 0.001**
Inattention	0.48 (0.39–0.56)	0.81 (0.73–0.88)	**0.008**
Disorientation	0.68 (0.59–0.76)	0.84 (0.76–0.90)	**0.005**
Hallucinations	0.50 (0.00–1.00)	0.40 (0.01–1.00)	0.811
Psychomotor agitation or retardation	0.24 (0.17–0.31)	0.36 (0.29–0.44)	**0.016**
Inappropriate speech or mood	0.39 (0.12–0.64)	0.38 (0.22–0.52)	0.91
Sleep–wake cycle disturbance	0.29 (0.25–0.32)	0.59 (0.51–0.68)	**< 0.001**
Symptom fluctuation	NA^a^	NA^a^	NA^a^

*Note:* *p* < 0.05 was considered statistically significant and is indicated in bold.

Abbreviations: ICDSC, intensive care delirium screening checklist; CI, confidence interval.

^a^ The hospital's electronic system automatically compares symptom fluctuation with the previous shift, eliminating the need for manual assessment.

### 5.2 Effect of Educational Intervention

A significant improvement in reliability for the ICDSC delirium assessment was observed following the intervention. Cohen's kappa value increased from 0.63 (95% CI: 0.57–0.70) to 0.71 (95% CI: 0.66–0.77; *p* = 0.04), whereas the overall percentage agreement rose from 76% to 82% (Table 1). As shown in Table 2, following the educational intervention, the most marked improvement was observed in altered level of consciousness (RASS), with the kappa statistic increasing from 0.52 to 0.80 (*p* < 0.001). Significant enhancements were also seen in inattention (*p* = 0.008) and disorientation (*p* = 0.005). Furthermore, improvements were noted in psychomotor agitation or retardation (from 0.24 to 0.36, *p* = 0.016) and sleep–wake cycle disturbance (from 0.29 to 0.59, *p* < 0.001).

No statistically significant change was observed for the item hallucinations and inappropriate speech or mood (*p* = 0.811 and 0.910, respectively), as these ratings consistently demonstrated high accuracy both before and after the intervention. For hallucination, AC1 values remained stable at 0.99 (95% CI: 0.98–1.00) across both time points. For inappropriate speech or mood, AC1 values were 0.95 (95% CI: 0.93–0.98) before and 0.89 (95% CI: 0.86–0.93) after the intervention, indicating consistently strong performance over time.

### 5.3 Patient Characteristics and Rating Disagreement

To examine factors associated with disagreements between nurse and expert ICDSC ratings, multivariable logistic regression analyses were conducted for the pre‐ and post‐intervention periods (Table 3). Before the intervention, older age was significantly associated with inaccurate nurse assessments, with odds ratios (ORs) of 1.70 for age > 65 (*p* = 0.022), 2.24 for age > 75 (*p* = 0.002) and 3.20 for age > 85 (*p* = 0.001). After the intervention, only the age > 85 category remained statistically significant (OR = 2.22, 95% CI: 1.21–4.07, *p* = 0.01). The use of physical restraints was consistently associated with rating disagreement in both phases: pre‐intervention OR = 4.25 (95% CI: 2.79–8.28, *p* < 0.001) and post‐intervention OR = 4.91 (95% CI: 1.92–12.54, *p* < 0.001). No significant associations were found for RASS score changes, mechanical ventilation or other age thresholds in the post‐intervention model.

**TABLE 3:** Logistic regression analysis of factors associated with inaccurate ICDSC assessments pre‐ and post‐intervention.

Patient‐related variables	Pre‐intervention		Post‐intervention	
	OR (95% CI)	*p*	OR (95% CI)	*p*
RASS change	1.23 (0.73–2.06)	0.425	0.73 (0.44–1.19)	0.205
Age > 65 y/o	1.70 (1.08–2.69)	**0.022**	1.65 (0.96–2.82)	0.07
Age > 75 y/o	2.24 (1.36–3.67)	**0.002**	1.21 (0.73–2.01)	0.454
Age > 85 y/o	3.20 (1.59–6.42)	**0.001**	2.22 (1.21–4.07)	**0.01**
Mechanical ventilation	0.82 (0.49–1.35)	0.429	1.44 (0.75–2.74)	0.274
Use of physical restraints	4.25 (2.79–8.28)	**< 0.001**	4.91 (1.92–12.54)	**< 0.001**

*Note:* *p* < 0.05 was considered statistically significant and is indicated in bold.

Abbreviations: CI, confidence interval; OR, odds ratio.

## 6 Discussion

The results of this study indicate that a structured, multimodal educational intervention significantly improved ICU nurses' performance in delirium assessment using the ICDSC, as evidenced by a notable increase in Cohen's kappa values across multiple assessment domains. This improvement may be attributed to the interactive and multimodal nature of the educational intervention, which provided nurses with both theoretical knowledge and practical experience in using the ICDSC, particularly through individualized, one‐to‐one bedside mentoring with real‐time feedback. In contrast to traditional verbal instructions and demonstrations by senior staff, the educational model in this study emphasized active, personalized bedside coaching, offering tailored learning opportunities that addressed specific challenges nurses encountered during routine assessments. Such hands‐on, context‐based training may be a key mechanism facilitating the effective translation of theoretical knowledge into clinical practice, thereby enhancing assessment performance [17, 18].

Despite these improvements, overall ICDSC performance did not reach the commonly accepted benchmark for optimal inter‐rater agreement, typically defined as Cohen's kappa > 0.8. Several factors may explain this limitation. A concerning upward trend in nurse turnover and vacancy rates in the post‐pandemic era has potentially compromised the performance level of ICDSC assessments. National statistics from the Nursing and Health Care Department [19] reveal a progressive increase in nurse turnover from 10.04% in 2018 to 12.61% in 2023, accompanied by a parallel rise in vacancy rates from 4.48% to 9.05%. This escalating workforce instability suggests a greater proportion of inexperienced ICU nurses who may possess insufficient expertise in delirium assessment. During the study period, our participating medical ICUs experienced a significant staffing transition, with 14 of 104 nurses being either newly recruited or transferred, representing a substantial 6.7% workforce change. Such personnel fluctuations likely contributed to inconsistencies in assessment quality and screening practices, particularly as newly integrated staff members may lack thorough familiarity with ICDSC criteria.

It is noteworthy that, following the intervention, five of the seven ICDSC items achieved ‘almost perfect’ agreement (κ or AC1 > 0.80). These included critical domains such as ‘Inattention’ and ‘Altered Level of Consciousness’, which are vital for the accurate detection of delirium. We acknowledge that further enhancements—through longer or repeated interventions—may be required to attain ‘excellent’ levels of agreement. Nevertheless, our findings demonstrate that this low‐cost, easily implemented educational intervention is feasible within ICU settings and significantly improves nurses' delirium assessment performance at the bedside. Thus, this structured, multimodal educational intervention is relevant and timely for hospitals wish to enhance ICU nurses' delirium assessment performance.

Furthermore, multivariable analysis indicated that although age‐related inaccuracies were observed before the intervention, nurses' assessment performance improved among patients aged 65–85 following training. However, patients over 85 years of age remained at higher risk of misclassification. This may reflect ongoing difficulties in recognizing delirium in the oldest‐old, likely due to subtler symptom presentation, communication barriers and underlying cognitive decline [20, 21]. These results suggest that although general educational efforts are beneficial, they may not fully address the unique challenges of assessing delirium in this particularly vulnerable population. Tailored approaches may be needed to improve assessment performance in very elderly ICU patients.

In addition to patient age, the use of physical restraints is also significantly associated with assessment inaccuracies. In the context of high nurse workload and restricted visitor access, restraints were often applied not in response to agitation, but pre‐emptively as a precautionary measure to prevent self‐extubation when nurses were not present at the bedside. This non‐behavioural rationale complicates the interpretation of psychomotor agitation, increasing the likelihood of inaccurate scoring in restrained patients using the ICDSC.

### 6.1 Limitation

Several limitations should be noted. First, although bedside nurses were blinded to the research nurse's assessments, the possibility of a Hawthorne effect cannot be excluded, as nurses may have altered their behaviour because of the awareness of being observed. Second, the effect of the educational intervention was assessed only shortly after its completion; therefore, the long‐term sustainability of the improvements in ICDSC assessment accuracy remains uncertain. Third, this study was conducted at a single centre, which may restrict the generalizability of the findings to other healthcare settings. Fourth, during the study period, 7 nurses left the ICUs and 7 new nurses joined. However, we did not implement any stratification or adjustment for these staffing changes, which may have had a potential impact on the results. Fifth, adherence to the intervention could not be tracked, as informed consent was waived for individual bedside nurses. We employed random and multi‐day sampling strategies each week to ensure that assessments from each bedside nurse had an equal opportunity to be included in comparison with the expert nurse. However, we acknowledge that some nurses may have completed more assessments than others, creating a potential bias that cannot be fully excluded.

### 6.2 Recommendations for Practice

To improve delirium assessments by ICU nurses, it is vital to integrate comprehensive educational interventions into training programmes. These should be implemented regularly and reinforced periodically to maintain nurse competencies.

Healthcare organizations should establish systematic audit‐and‐feedback systems to evaluate assessment quality and provide personalized coaching for inconsistencies. Future research should explore the longevity of educational interventions and the use of digital decision‐support tools and simulations to improve assessment accuracy and inter‐rater agreement.

## 7 Conclusions

This study demonstrates that targeted, multimodal educational interventions can meaningfully enhance the performance of ICU nurses' delirium assessments using the ICDSC. However, residual inconsistencies underscore the need for ongoing educational reinforcement and increased sensitivity in applying certain ICDSC items. In our setting, physical restraints are often used pre‐emptively because of high workload, complicating the interpretation of ICDSC item 5, which links hyperactivity to the need for sedation or restraints. This rigid application may lead to misclassification. Our findings indicate a need to refine the interpretation and application of specific items across various clinical and cultural contexts. Future research should explore the long‐term effects of such interventions and investigate additional strategies for enhancing delirium detection in critically ill patients.

## Ethics Statement

The study was approved by the National Taiwan University Hospital Research Ethics Committee (202301153RINB) and registered at the Clinical Trials Registry (Trial No.: NCT06054828).

## Consent

The authors have nothing to report.

## Conflicts of Interest

The authors declare no conflicts of interest.

## Data Availability

The data that support the findings of this study are available from the corresponding author upon reasonable request.
